# Can the Body Mass Index Predict Varicocele Recurrence Post-Intervention?

**DOI:** 10.7759/cureus.14892

**Published:** 2021-05-07

**Authors:** Saad Abumelha, Abdullah Alkhayal, Khalid Alrabeeah, Ahmed Khogeer, Ghassan I Alhajress, Mohammed Alabdulsalam, Yousof Al Zahrani

**Affiliations:** 1 College of Medicine, King Saud bin Abdulaziz University for Health Sciences, Riyadh, SAU; 2 Division of Urology, Department of Surgery, Ministry of National Guard - Health Affairs, Riyadh, SAU; 3 College of Medicine, King Abdullah International Medical Research Center, Riyadh, SAU; 4 College of Medicine, King Saud Bin Abdulaziz University for Health Sciences, Riyadh, SAU; 5 Department of Urology, King Faisal Specialist Hospital and Research Center, Jeddah, SAU; 6 Department of Radiology, Ministry of National Guard - Health Affairs, Riyadh, SAU

**Keywords:** andrology, embolization, varicocele, varicocelectomy

## Abstract

Purpose

In this study, we investigated the association between the body mass index (BMI) and varicocele recurrence post-intervention in our local Saudi population. We also explored the association between recurrence and other predictors such as age, laterality, indication for surgery, type of intervention, clinical grade, testicular delivery, and method of ligation.

Methods

We conducted a retrospective cohort study, including all patients who had microscopic varicocelectomy surgery or radiographic embolization for varicoceles over a five-year period. The data included demographic information and intervention-related variables. Descriptive and analytical statistics were used to analyze the data.

Results

We included 147 patients who had microscopic varicocelectomy surgery or radiographic embolization. We categorized the patients according to their BMI as underweight, normal, overweight, and obese. We found no statistical association between any BMI group and the recurrence of varicocele (P>0.05). However, there was a significant association between the clinical grade and recurrence (P<0.05).

Conclusion

This study did not show any significant correlation between height, weight, BMI, and varicocele recurrence after an intervention. The only predictor of varicocele recurrence was the clinical grade.

## Introduction

A varicocele is an abnormal dilation of the venous pampiniform plexus of the spermatic cord. It is found in 15% of adolescent males and more than 30% of infertile men [1,2]. Though most cases are asymptomatic, 2-10% present with a dull, stabbing pain [1]. Most of the patients seek medical attention due to infertility concerns [1]. Although many patients with a varicocele are fertile, the association with abnormalities in concentration, morphology, and motility of sperms has been reported [1].

Multiple modalities are available for the treatment of varicocele. Retroperitoneal and laparoscopic operations aim to isolate the internal spermatic vein proximally near the internal inguinal ring [3]. Microsurgical inguinal and sub-inguinal approaches are the current standard of treatment as they allow access to the gubernacular and external spermatic veins [3]. The use of the operating microscope significantly decreases the risks of postoperative hydrocele, testicular atrophy, and recurrence. In addition, emerging radiographic occlusion techniques is a viable option in certain patients and are successful in 75% to 90% of the cases [3].

As more modalities are becoming available, practitioners are required to offer the patients evidence-based counseling regarding the risks related to the interventions. Recurrence is a complication frequently explored in the literature; however, few papers focus on the predisposing pre-intervention factors. The incidence of recurrence post-intervention is between 0.6% and 28%, depending on the chosen modality [4-7]. Additional studies are required to clarify whether patient-related and procedure-related factors predispose patients to a recurrence.

In the current study, we aimed to assess the association between body mass index (BMI) and varicocele recurrence post-intervention. We also explored the association between varicocele recurrence and other predictors such as age, clinical grade, laterality, reason for surgery, and type of intervention.

## Materials and methods

The study design was a retrospective cohort study. The patients' medical records were reviewed, and the data were collected using a data collection form. All patients who underwent microscopic varicocelectomy surgery or radiographic embolization of a unilateral or bilateral varicocele from January 1, 2016, to December 31, 2019, in our tertiary care center were included in the study. All the patients diagnosed with varicocele but not with microscopic varicocelectomy surgery or radiographic embolization were excluded. We also excluded patients who had previous interventions in other centers, and patients lost to follow-up post-intervention. 

The record of the first visit to the clinic was used to report the clinical examination. We used the measurements of height and weight recorded in the clinic preoperatively. The BMI score was calculated using the formula: weight in kilogram divided by the square of the height in meters. All the patients who received a surgical intervention had a sub-inguinal microscopic varicocelectomy and operated on as day surgery cases by two surgeons with fellowships in andrology. We reviewed the operative notes to obtain the data. Varicocele recurrence was defined by the clinical examination findings at least three months after the intervention. Follow-up ultrasound studies were requested for patients with a clinical recurrence. The radiologist reviewed all the ultrasound findings to standardize reporting between patients.

Descriptive and analytical statistics were conducted. The Statistical Package for the Social Sciences (SPSS) software for Windows ver. 24 (IBM Corp., Armonk, NY, USA) was used for data analysis. The mean and standard deviation (SD) were calculated for quantitative variables such as age, weight, height, BMI, veins ligated, first ultrasound study maximum diameter, and follow-up ultrasound study maximum diameter. Frequency and percentage were calculated for the qualitative variables. A one-way analysis of variance (ANOVA) test was used to compare the recurrence of varicocele and the quantitative data (BMI and age). A Chi-square test was used for both dependent and independent qualitative variables.

The study was approved by the Institutional Review Board at King Abdullah International Medical Research Center, Riyadh, Saudi Arabia (IRB#: RYD-20-419812-174023). The manuscript has been read and approved by all the authors, and each author indicated that this manuscript represents appropriate and honest research.

## Results

In total, 147 patients who underwent surgery or embolization from January 1, 2016, to December 31, 2019, after applying our exclusion criteria, were included. Table 1 displays the demographic information of the sample.

**Table 1:** Sample characteristics

Variable	Minimum	Maximum	Mean	Standard deviation
Age	16	67	35.84	1.03
Weight (kg)	43	170	84.5	2.10
Height (cm)	153	190	171	6.80
BMI (kg/m^2^)	16	45.92	28.15	6.11

Age was categorized as less than 25 years, 25-45 years, and above 45 years. The BMI was categorized according to the National Institutes of Health Clinical guidelines as underweight (<18 kg/m^2^), normal (18-25 kg/m^2^), overweight (25-30 kg/m^2^), and obese (>30 kg/m^2^).

For the majority of the sample (n=91, 61.9%), the indication for surgery was infertility, followed by symptomatic causes (n=54, 36.7%) and occupational causes (military pre-employment medical examinations; n=2, 1.4%). The majority (n=108, 73.5%) of the varicoceles were left-sided, two (1.4%) right-sided, and 37 (25.2%) bilateral. A small proportion (n=23, 15.6%) had grade I varicoceles, 80 (54.4%) grade II varicoceles, and 44 (29.9%) grade III varicoceles.

Regarding the type of intervention, the majority (n=122, 83%) had surgical intervention, while 25 (17%) had embolization. Additional details of the surgical intervention are shown in Table 2. Of the embolization group, 23 (15.6%) were embolized with coils and sclerotherapy, 1 (0.7%) with glue, and 1 (0.7%) with coils only. At follow-up, 134 (91.2%) of the patients did not have a recurrence, with 13 (8.8%) having a recurrence.

**Table 2:** Surgical intervention details *Delivery of testis to the inguinal wound to assess for the gubernacular vein and ligate it if present.

	Number of patients	(%)
Method of ligation		
Ties	68	46.3
Clips	54	36.7
Delivery of testis^*^		
Delivered	54	36.7
Not delivered	68	46.3

Of the sample, 111 (75.5%) had reflux on ultrasound preoperatively. In terms of the ultrasound grading, 75 (51%) of the sample had grade I varicocele, 38 (25.9%) grade II, and only 8 (5.4%) grade III. Regarding the patients with a suspicion of clinical recurrence at their follow-up consultation, 21 (14.3%) had reflux on ultrasound, and 1 (0.7%) no reflux on ultrasound. In addition, 17 (11.6%) with a recurrence on the follow-up ultrasound had grade I varicocele, 3 (2%) grade II, and only 1 (0.7%) grade III.

Analytical statistics were done to determine any statistical association between multiple factors and varicocele recurrence. There was no significant association between the BMI or age groups and varicocele recurrence (P>0.05). There was also no statistical association between the reason for surgery and the recurrence of varicocele (P>0.05), between laterality and recurrence of varicocele (P>0.05), or the type of intervention and varicocele recurrence (P>0.05). However, the association between the clinical grade and recurrence was statistically significant (P<0.05; Table 3).

**Table 3:** Association between variables and clinical recurrence

Variable	Recurrence (N = 13)	No recurrence (N = 134)	P-value
BMI group			
Underweight	0 (0%)	5 (3.4%)	0.37
Normal	6 (4.1%)	36 (24.5%)	
Overweight	2 (1.4%)	43 (29.3%)	
Obese	5 (3.4%)	50 (34%)	
Age group			
Below 25 years	1 (0.7%)	22 (15%)	0.14
25–45 years	8 (5.4%)	96 (65.3%)	
More than 45 years	4 (2.7%)	16 (10.9%)	
Type of intervention			
Surgery	10 (6.8%)	112 (76.2%)	0.54
Embolization	3 (2%)	22 (15%)	
Laterality			
Left	12 (8.2%)	98 (66.7%)	0.13
Right	1 (0.7%)	36 (24.5%)	
Clinical grade			
I	1 (0.7%)	22 (15%)	0.03
II	4 (2.7%)	76 (51.7%)	
III	8 (5.4%)	36 (24.5%)	
Reason for surgery			
Infertility	10 (6.8%)	81 (55.1%)	0.48
Symptomatic	3 (2%)	51 (34.7%)	
Occupational	0 (0%)	2 (1.4%)	

## Discussion

Varicocele is a frequent problem encountered by urologists in clinical practice, and recurrence is a known complication following surgery. The pathophysiology underpinning the recurrence depends on the surgical technique. For a subinguinal varicocelectomy, recurrence is secondary to the dilation of missed smaller internal spermatic veins [8]. In the current study, at the follow-up consultation, 13 patients (8.8%) had a recurrence. The mean age of the sample was 35.8 years, which is higher than the mean age in the literature [9-11], probably due to our center’s large andrology service. The results indicated no association between age and clinical recurrence, which is consistent with the literature.

The BMI ranged from 16 kg/m^2^ to 45.9 kg/m^2^ with a mean BMI of 28.15±6.11 kg/m^2^. This is higher than the BMI reported in the literature related to this association, including 23.5 kg/m^2^ [9], 22.8 kg/m^2^ [3], and 25.1 kg/m^2^ [10]. We found no significant association between BMI or age and varicocele recurrence, although the overall higher BMI of the population could be a factor. Other studies, such as Li et al. [11], concluded that a BMI lower than 25 kg/m^2 ^significantly increases the recurrence rate after repair. Míguez et al. [12] reported an association between left renal vein entrapment and varicocele recurrence, especially in patients with a low BMI. In addition, Rotker and Sigman et al. [13] reinforced the notion that varicocele recurrence was associated with tall, thin stature. Further research related to the risk of recurrence in low BMI patients is warranted.

With the initial ultrasound grading, 75 (51%) of the sample had grade I varicocele, 38 (25.9%) grade II varicocele, and only eight (5.4%) grade III varicocele. The results indicated a significant association between clinical grade and recurrence (P<0.05). In other words, an advanced grade of varicocele was associated with a higher recurrence rate, which is supported in the literature. We recommend that patients with higher grades of varicocele be counseled regarding the risk of recurrence, and the choice of intervention should be limited to the modalities with the lowest recurrence [3,9,10].

A systematic review by Fazeli et al. [8] reported that microsurgical inguinal or sub-inguinal varicocelectomy had the lowest recurrence rate compared to other techniques, such as macroscopic, retroperitoneal high ligation, or radiological embolization. This is possibly secondary to missed smaller internal spermatic veins, with using a macroscopic technique, the inability to ligate either the cremasteric vessels or the external spermatic veins with retroperitoneal techniques, or recanalization in case of embolization. The review concluded that a significant reason for recurrence is a failure to ligate branched spermatic veins. The current study showed no statistical association between the type of intervention and the recurrence of the varicocele (P>0.05).

There are several limitations to our study. First, the sample included only the patients attending one tertiary care hospital, primarily treating persons with a military background and their immediate family, which may not be applicable to the general wider population. Second, there was a high rate of loss to follow-up. From our experience with our patients, this is due to a tendency to avoid going to the hospital unless there is an active problem. Third, the sample size of the group with a recurrence was relatively small. Studies with a larger sample size are required to explore these findings in-depth.

## Conclusions

The results of the current study indicated a significant association between an advanced grade of varicocele and recurrence; however, the small sample size and retrospective design limited this study. Though the study did not show a significant association between height, weight, BMI, and varicocele recurrence after an intervention, it highlighted the necessity for further studies in this area, to provide insight into the pathophysiology of varicocele and improve patient care.
